# A case of asystole from carotid sinus hypersensitivity during patient positioning for thyroidectomy

**DOI:** 10.1186/s12871-016-0255-5

**Published:** 2016-10-06

**Authors:** Emmanuel Lilitsis, Alexia Papaioannou, Aikaterini Hatzimichali, Konstantinos Spyridakis, Sofia Xenaki, George Chalkiadakis, Emmanuel Chrysos

**Affiliations:** 1 Department of Anaesthesiology, University Hospital of Crete, Heraklion, Crete Greece; 2 Department of General Surgery, University Hospital of Crete, Heraklion, Crete Greece

**Keywords:** Thyroidectomy, Hyperextension, Position, Asystole

## Abstract

**Background:**

We present a case of a patient with multinodular goiter disease who suffered asystole during head hyperextension for surgical positioning on the operational table.

**Case Presentation:**

Manipulation of carotid sinus may trigger bradycardia or even asystole even in patients without prior history of carotid sinus hypersensitivity. The time proximity between patient positioning and asystole, the late responsiveness to atropine, the immediate increase of heart rate after head elevation and the lack of any other trigger factor or prior history support the hypothesis of carotid sinus syndrome.

**Conclusions:**

Head hyperextension during surgical positioning is not only responsible for jeopardizing blood flow to spinal cord and brainstem but may trigger reflexes, as well, even in patients without prior neck pathology.

## Background

Supine is the most commonly used position for surgery. The effects of the position on the physiology of the patient are expected to be minimal and well tolerated. Yet access to surgical site often demands the placement of a patient in an unnatural position risking patients’s wellbeing. Positioning may provoke direct anatomical damage like pressure ulcers and nerve damages or alters the patient’s physiology mainly by affecting the cardiovascular and pulmonary homeostasis [1, 2].

Thyroidectomy has been associated with a variety of serious complications, such as hematoma, recurrent laryngeal nerve damage, tracheomalacia, and accidental loss of parathyroids resulting in hypocalcemia [3]. Head and neck surgeries like thyroidectomy, tonsillectomy and carotid endarterectomy necessitates the hyperextension of the head giving access to surgical site. Indeed there are case reports of patients suffering tetraplegia after parathyroidectomy as a result of hyperextension of head indicating that this extreme positioning may jeopardize blood flow to carotid and vertebral arteries leading to brainstem and cervical spine ischemia especially in older patients with degenerative cervical spine diseases [4]. Less known complication of head extension is triggering cardiovascular reflexes like carotid sinus reflex. It is presented an interesting case of a patient who suffered sudden cardiac asystole after positioning for thyroidectomy on the surgical table.

## Case presentation

A 56 year old woman of the Caucasian race, was scheduled for thyroidectomy due to multinodular goiter according to u/s assessment. She was euthyroidic with normal TSH, fT3 and fT4 under levothyroxine. Other medical history included arterial hypertension under 150 mg irbesartan, palpitations under 2.5 mg nebivolol. She was a current smoker with 40 p/y and a BMI 22.6 kg/m2 (52 kg). Prior surgery was a tonsillectomy 40 years ago for which she had no uneventful information about the surgery or the anesthesia itself. During clinical examination and history she denied any coronary artery disease symptoms, stroke, syncope, near-syncope, dizziness, unexplained falls and she had good physical status. She refused having allergies. During the airway assessment it was observed normal range of motion for head flexion and extension without any symptoms. Presurgical ECG was examined by a cardiologist and revealed normal sinus rhythm with normal PQ, QRS intervals with a HR of 70/min. Vital signs on hospital admission was BP 125/75 mmHg, HR 78/min, SpO2 98 %. No pathological signs were detected either in the thoracic x-ray or the lab blood tests - normal complete blood count, blood clotting test and basic electrolyte, renal and liver biochemistry. As far as the goiter is concerned, an ultrasound of the neck was performed which revealed an enlarged thyroid gland was. In details the right lobe sized 1.24 × 1 × 26 × 3.47 cm and the left lobe sized 0.83 × 0.93 × 2.58 cm. As pointed in the ultrasound, in the lower surface of the right lobe it was detected a nodule sized 1.62 × 1.37 cm with peripheral hematosis as well as two more cystic nodules sided 1.25 × 1 × 15 cm each. The goiter did not press the carotid vessels and was definitely not submersible. The goiter surely has taken part in the compression of the carotid sinus when the neck was extended, though it was not the absolute reason of the asystole.

In accordance with literature guidelines about the administration of perioperative b-blocker [5, 6], the patient received nebivolol the day of the surgery along with p.o. 1.5 mg of bromazepam for premedication, ranitidine 150 mg, domperidone 10 mg and inhalation therapy with salbutamol-impratropium/budesonide since she was a current smoker. Irbesartan was witheld. The patient was connected to the monitor and her vital signs were BP 132/68 mmHg (non invasive BP), HR 65/min, SpO2 98 % on room air (FiO2 21 %) and respiratory rate 17/min. After intravenous access was managed and the patient was properly preoxygenated general anesthesia was induced with 100 μgr of fentanyl, 40 mg of lidocaine, 140 mg of propofol and 12 mg of cisatracurium. Approximately 3 min later the patient was easily intubated and connected to the anesthesia machine. General anesthesia was maintained with 40 % of oxygen and 1.9 % of sevoflurane targeting 1 MAC. Vital signs after the induction were BP 102/55 mmHg, HR 54/min, SpO2 97 %. Volume control ventilation was used targeting an end expiratory CO2 around 35 mmHg. The patient’s HR varied from 52 to 59/min with systolic BP measurements around 94–108 mmHg. The patient then was positioned for thyroidectomy, a roll was placed behind her shoulders so her torso would be elevated exposing the neck while the head was extended and positioned on a soft silicon pillow.

Within few seconds the head was placed monitor’s HR alarm went on for bradycardia, 38/min. Immediately a pre filed syringe with 1 mg atropine was intravenously flushed. Heart rate deteriorated further reaching 10/min and finally a complete asystole was recorded on the monitor with no carotid pulse palpable. Cardiopulmonary resuscitation was immediately offered and a nurse supplied the second anesthesiologist with a pre filled syringe of 1 mg adrenaline. Asystole was still ongoing aproximately 45 s with no visible response to the already injected atropine. The first anesthesiologist performing CPR suspected that head extension was a possible trigger factor and raised the patient’s head to a neutral position before adrenaline was infused. Immediately after the patient’s head was raised monitor showed a heart rate of 79/min which after few seconds raised to 113/min. Adrenaline was withheld since the patients had a palpable carotid pulse and the next BP measurement was 157/78. The time proximity of asystole with the head extension gave the impression that the specific positioning created a tension to possibly both the carotid sinuses explaining the nonresponsiveness to atropine and why normal HR was immediately achieved only by elevating the head.

Since the episode had a small duration it was decided that the surgery would be still continued. The patients’s head was then placed on an upper position using a higher pillow and thyroidectomy was performed uneventfully. At the end the patient was awakened and extubated with no neurological deficit. Basic biochemistry with troponin and postsurgical ECG revealed no pathological findings. In the post anesthetic care unit the patient was informed about the episode and again was asked for any kind of symptoms related to carotid sinus reflex-syndrome. She refused them all. She was given a signed paper about what happened and was strongly advised to inform the anesthesiologist at any possible future surgeries. The patient was referred to a cardiologist for further assessment.

## Discussion

Positioning the patient is definitely an important aspect of any head and neck surgery. Adequate exposure of the neck is essential. Thus the typical “Rose” or “Kocher” position is used in any thyroidectomy surgery. In details, the patient is placed flat (supine) on the operating table and the neck extended to pull the thyroid from the root of the neck. A sponge doughnut is placed under the occiput for adequate head support and a special pillow named “Dunhill pillow” supports the shoulder blades. The whole operating table is titled 5–10° feet-down in order to reduce venous pressure in the neck [7, 8].

The carotid sinus as a baroreceptor is responsible for maintaining blood pressure within narrow limits. The carotid sinus is innervated by the nerve of Hering, a branch of cranial nerve IX (glossopharyngeal nerve). The afferent part of the reflex synapses in the nucleus tractus solitarii (NTS) located in the medulla oblongata of the brainstem. The NTS regulates the activity of autonomic nervous system through the hypothalamus [9]. Carotid sinus syndrome is defined as cardiac syncope during carotid sinus massage of 10s duration. There are two types known. Cardioinhibitory type when sinus manipulation provokes 3 s asystole and the vasodepressor type when systolic blood pressure falls 50 mmHg. Mixed forms show both features. Carotid sinus hypersensitivity (CSH) does not necessarily coexist with carotid sinus syndrome (CSS) [10]. CSH was previously considered a rare cause of syncope. It is uncommon in the young but the incidence increases with age >50. Recent data suggests that the incidence in elderly with unexplained falls may be considerably higher than in general elderly population even reaching 60 % [10, 11].

Carotid sinus is one of the many neurally mediated reflex regulators of blood pressure. There are some hypotheses that have been proposed and could be related to our case: the denervation of the sternocleidomastoid hypotheses and the vascular hypotheses. There are studies supporting that patients with carotid sinus syndrome have altered sternocleidomastoid electromyography than healthy controls. A hypotheses was made that sternocleidomastoid has proprioceptive sensitivity which acts as a negative feedback to carotid sinus reflex arc. When the sternocleidomastoid becomes denervated with age this proprioceptive inhibitory effect is diminished and the carotid sinus loses this regulation [12, 13]. We believe that this theory may explain why the reflex was provoked in our patient during head hyperextension. Both carotid sinuses were possibly triggered but possibly with no negative input from the sternocleidomastoids the response was exaggerated leading to sudden arrest. The vascular hypothesis regarding the pathophysiology of the reflex relies on the clinical observations that it is associated with atherosclerotic diseases and increases in frequency with age. Since the sinus reflex is triggered by tension receptors, any loss of arterial distensibility with age and with atherosclerosis may reduce neural output of the afferent limb of the reflex loop. As a result the expected compensatory effect is baroreflex efferent hypersensitivity and enhancement of the reflex [14–16]. Our patient had long standing arterial hypertension and was current smoker, both known risk factors for atheroscleroris.

Study by Gustavo et al. assessing 502 patients with a history of unexplained syncope or falls found that the use of negative chronotropic drugs are associated with a risk ratio 2.18 of developing cardioinhibitory response of carotid sinus hypersensitivity [16]. B blockers not only block sympathetic nervous system leaving unopposed the activity of vagus nerve but along with digitalis and methyldopa enhance the sensitivity of carotid sinus (http://emedicine.medscape.com/article/153312). Fentanyl is also known to cause bradycardia through direct action on micro opioid receptors on cardiac vagal neurons [17] Propofol blocks sympathetic nervous and inhibits GABAergic neurotransmission to cardiac vagal neurons, which would evoke a decrease in heart rate [18]. Both drugs were used during induction of general anesthesia.

As far as the compression or the invasion of the tumor is concerned, unexplained abrupt episodes of bradycardia and intermittent heart block should alert the physician of the possibility of carotid sinus compression by a neck mass in patients with head and neck cancer. As described in the article of Mehta et al. 2014, in most cases of CSS (Carotid Sinus Syndrome), no obvious precipitating cause can be identified. Earlier stated trigger mechanisms include head and neck movement, hyperextension of the neck, constricting neckwear (such as collars) and shaving to name a few. Any pathology close to the carotid sinus such as carotid body tumors, local tumors such as thyroid cancers, cervical lymphadenopathy, and internal carotid artery aneurysms can also cause CSS. There are many theories to explain CSS in patients with head and neck cancer. Muntz and Smith in 1983 postulated that a tumor located near the Carotid Sinus or it’s nerve could cause a permanent depolarization of its axons with an increased tendency of adjacent uninjured axons to fire impulses causing signs and symptoms of CSS [19]. Radiation therapy of the carotid sinus has often been used to treat malignancies of the head and neck region, but some case series have described it as a cause of CSH (by post-irradiation fibrosis) by a mechanism similar to the one just described [20]. Wallin and Westerberg suggested that syncope in these patients is caused by abnormal impulses that are rerouted via the pharynx to the dorsal motor nucleus of vagus, causing excitation of the vagus [21]. Glossopharyngeal nerve invasion by a tumor can also cause neuralgia without any disease in the carotid sinus area, termed glossopharyngeal neuralgia [22]. Lesions at the cerebellopontine angle can cause cardiovascular symptoms similar to CSS. In spite of these theories, the pathophysiology of CSS due to head and neck cancer is yet to be fully understood [23].

The incidence of cardiac arrest perioperatively due to carotid sinus sensitivity is rare and most cases describe patients being treated for head and neck pathology [24, 25]. We found one case report describing a patient suffering cardiac arrest twice during head positioning for thyroidectomy but this patient was previously treated with radiotherapy which provoked the formation of stiff scar tissue and altered the physiology of the carotid sinus [26]. No case report before described cardiac arrest during patient positioning for thyroidectomy without any pathology like prior surgery or radiotherapy.

## Conclusion

Head hyperextension during head and neck surgery is known to cause neurological or other complications to the patients. There are studies suggesting to position the patient before general anesthesia in order to find the “comfort zone” and avoid symptoms like arm numbness or headache. Head hyperextension during surgical positioning is not only responsible for jeopardizing blood flow to spinal cord and brainstem but may trigger reflexes, as well, even in patients without prior neck pathology. With this article we suggest vigilance even in healthy patients requiring head extension due to possible trigger of carotid sinus reflex and cardiac asystole. Doctors should be aware that even the use of atropine may not reverse the reflex and bradycardia and should alleviate the pressure on the carotid sinus by flexing the head of the patient.

## Abbreviations

BMI: Body mass index
BP: Blood pressure
CPR: Cardio pulmonary resuscitation
CSH: Carotid sinus hypersensitivity
CSS: Carotid sinus syndrome
ECG: Echocardiogram
HR: Heart rate
NTS: Nucleus tractus solitarii
